# Sex differences in relation patterns between health-related quality of life of older adults and its correlates: a population-based cross-sectional study in Madeira, Portugal: Cite this article: Gouveia BR, Ihle A, Kliegel M, Freitas DL, Gouveia ÉR. (2018) Sex differences in relation patterns between health-related quality of life of older adults and its correlates: a population-based cross-sectional study in Madeira, Portugal. *Primary Health Care Research & Development* page 1 of 5. doi: 10.1017/S1463423618000233

**DOI:** 10.1017/S1463423618000233

**Published:** 2018-09-13

**Authors:** Bruna R. Gouveia, Andreas Ihle, Matthias Kliegel, Duarte L. Freitas, Élvio R. Gouveia

**Affiliations:** 1 Saint Joseph of Cluny Higher School of Nursing, Funchal, Portugal; 2 Health Administration Institute, IP-RAM, Secretary of Health of the Autonomous Region of Madeira, Funchal, Portugal; 3 Center for the Interdisciplinary Study of Gerontology and Vulnerability, University of Geneva, Geneva, Switzerland; 4 Madeira Interactive Technologies Institute, University of Madeira, Funchal, Portugal; 5 Department of Psychology, University of Geneva, Geneva, Switzerland; 6 Department of Physical Education and Sport, University of Madeira, Funchal, Portugal; 7 Department of Mathematical Sciences, University of Essex, Colchester, UK

**Keywords:** Health-related quality of life, older adults, sex differences

## Abstract

A population-based cross-sectional study aimed to examine sex differences in health-related quality of life (HRQoL) of older adults, and investigate whether the relation patterns between HRQoL and its correlates differed between sexes. A stratified proportional and representative sample included 802 volunteers, aged 60–79. HRQoL (36-item Short Form Health Survey), functional fitness (Senior Fitness Test), physical activity (PA) (Baecke questionnaire), demographic information and health features (questionnaires) were assessed. Men showed significantly higher HRQoL (*P*<0.001). Body mass index, body strength, aerobic endurance, PA, depressive symptoms, falls, and living alone were significantly related to HRQoL. With sex as moderator, these relations were not significant, except for PA (*β*=0.12, *P*=0.004). A significant interaction of sex with PA on HRQoL (*β*=0.08, *P*=0.037) was found, indicating that this relation was higher in men. A similar relation pattern was found for HRQoL physical component. HRQoL and its correlates differed between sexes, demanding a sex specific approach to promote HRQoL.

## Introduction

Quality of life has increasingly been discussed as an important outcome in gerontological research, as it plays an important role in enhancing successful aging (Choi *et al*., 2017). As a complex concept, it has been described in many constructs (Rejeski and Mihalko, 2001; Hambleton *et al*., 2009; Gouveia *et al*., 2017). In the present research, we focused on health-related quality of life (HRQoL). Within this perspective, attention is given to individuals’ physical and mental health (MH) perceptions and their dimensions, namely physical functioning, physical role functioning, bodily pain (BP), general health (GH) perceptions, vitality (VT), social role functioning, emotional role functioning, and MH (Ware and Sherbourne, 1992).

It is well established that many factors affect HRQoL in old age. On the one hand, a negative relation to HRQoL has been found for obesity (Dale *et al*., 2013; Giuli *et al*., 2014; Glintborg *et al*., 2014; Dhana *et al*., 2016), depressive symptoms (Sivertsen *et al*., 2015), social isolation (Hawton *et al*., 2011), falls (Stenhagen *et al*., 2014; Thiem *et al*., 2014). On the other hand, a positive association with HRQoL has been found for physical activity (PA) (Wanderley *et al*., 2011; Dale *et al*., 2013; Olsson *et al*., 2015; Haider *et al*., 2016) and functional fitness (Takata *et al*., 2010; Olivares *et al*., 2011; Wanderley *et al*., 2011; Haider *et al*., 2016). Although HRQoL has been addressed in all these studies, a better understanding of its relationships is still needed. Bearing in mind the crucial role of HRQoL in old age and the need for tailored approaches to its enhancement, the possibility that the relationship pattern of HRQoL to its correlates may depend on individual factors should be considered. However, so far, evidence on this issue is scarce. For example, while sex differences in older adults’ HRQoL (with men reporting higher HRQoL) and related inequalities have been extensively discussed in prior research (Skar *et al.*, 2014; Katz and Calasanti, 2015; Olmedo-Alguacil *et al*., 2016), such sex differences, remain to be further explained. Yet, in this regard, evidence on sex differences in the relation between HRQoL and its correlates in old age is insufficient so far (Orfila *et al*., 2006; Noh *et al*., 2015; Hajek *et al*., 2016; Hart, 2016).

Therefore, addressing this open issue, the present study sets out to examine sex differences in HRQoL of older adults, and to investigate whether the relation patterns between HRQoL and its correlates [body mass index (BMI), body strength, aerobic endurance, PA, falls, depressive symptoms, and living alone] differed between sexes between men and women.

## Methods

### Study design and participants

The present population-based cross-sectional study included a stratified proportional sample of volunteer older adults: 802 participants, 401 males and 401 females, from the Autonomous Region of Madeira, Portugal. Sex and age cohort served as stratification variables. Mean age of the overall sample was 69.8 years (SD=5.6), similarly distributed over the two subgroups (see Table 1). The sample size was determined following an *a priori* power analysis, which indicated that, to detect a small relation of *r*=0.15, with a two-tailed *α* probability of 0.05 and a power of 0.99, the sample size would need to comprise 806 individuals.

**Table 1:** Descriptive statistics and sex differences in health-related quality of life, health-related parameters, and lifestyle variables

	Men (mean±SD) (*n*=401)	Women (mean±SD) (*n*=401)	*P*
Age (years)	69.9±5.6	69.8±5.6	0.771
SF-36 total score (0–800)	592.2±123.8	505.0±149.0	<0.001
PC (0–400)	286.8±75.5	237.4±87.6	<0.001
MC (0–400)	305.4±61.2	267.6±77.5	<0.001
BMI (kg/m^2^)	28.8±3.9	30.3±4.6	<0.001
ACT (*n*)	16.5±3.8	16.1±4.3	0.175
MWT6 (min)	521.2±113.3	458.6±110.7	<0.001
PA total score (3–15 units)	7.3±1.3	7.3±1.2	0.680
Depression (%)	13.1	33.8	<0.001
Fallers (%)	23.6	48.7	<0.001
Liv. alone (%)	7.0	27.4	<0.001

SF-36=36-item Short Form Health Survey; PC=physical component; MC=mental component; BMI=body mass index; ACT=arm curl test; MWT6=6-min walk test; PA=physical activity; Liv. alone=living alone. *P* values are results from independent samples *t*-tests comparing the mean scores in men versus women and of *χ* ^2^ tests comparing sample proportions.

Inclusion criteria were as follows: (1) being community dwelling; (2) age between 60 and 79 years; and (3) to be able to walk independently. Exclusion criteria were as follows: (1) any medical contraindications to sub-maximum exercise (American College of Sports Medicine, 2006); and (2) inability to understand and follow the assessment protocol of the study, explained in Portuguese language.

Recruitment strategies included communications about the project in day-care and social centers, cultural and sports clubs and associations, and residential and public places (eg, open markets, municipal gardens, and churches). The study was also advertised in the local newspaper, radio, and television. Volunteers were then invited to the university laboratory, for the assessments.

### Data sources

HRQoL was assessed using the 36-item Short Form Health Survey (SF-36; Ware and Sherbourne, 1992; Portuguese version validated by Ribeiro, 2005). The SF-36 includes eight dimensions: physical functioning (PF), role physical (RP), BP, GH, VT, social functioning (SF), role emotional (RE), and MH. Scores of each dimension range from 0 to 100, with higher scores indicating better HRQoL. Two major components can be obtained by sum scores: (1) physical component (PF+RP+BP+GH) and (2) mental component (VT+SF+RE+MH), with scores ranging from 0 to 400. In this study, the Cronbach’s *α* coefficient was 0.728 and 0.729 for the physical and the mental component, respectively. The total SF-36 score can be obtained by a sum score: physical component+mental component, with scores ranging from 0 to 800.

PA was assessed using the Baecke questionnaire (Baecke *et al*., 1982). The questionnaire includes 16 questions, classified into three specific domains: PA at work/household activities, sports, and leisure time. It provides a measure of habitual PA which is the sum of these three specific domains. Numerical coding for most response categories varied from 1 to 5 (Likert scale) ranging from never to always or very often. A detailed description of the scoring procedures for calculation of habitual PA and its sub-component categories (PA at work, sports, and leisure time) is provided by the author. In this study, interviews were taken alternately by four different researchers over the course of data collection. Previously, intra-class correlation coefficients were calculated to determine the test–retest reliability of the questionnaire in a pilot study involving 32 males and 59 females (68.3; SD=7.6 years). Over an interval of one week, correlations ranged between 0.83, 0.85, and 0.85 for the work, sports, and leisure-time indices, respectively. Our reliability scores for work and sports PA were similar to those obtained by Baecke *et al*. (1982) (0.88, 0.81) and higher for leisure time index (0.74). The validity of this questionnaire has been also established by Ono *et al*. (2007) for this population using digital pedometry and uniaxial accelerometry (correlations ranged from 0.30 to 0.49).

Functional fitness components were assessed: body strength (using the arm curl test) and aerobic endurance (using the 6-min walk test; Rikli and Jones, 2013). Validity estimates have been assessed by the authors of the battery. BMI was calculated from weight and height (kg/m^2^). All tests were developed in the same laboratory and conditions, following the established protocol. All assessors undertook 5 h of theoretical explanation, followed by practical training (10 h). In a pilot study, 50 older adults were assessed. Test–retest correlation (*R*) were between 0.75 and 0.90.

Individual health (history of depression and falls during the last year) and demographic information (age, living alone) were obtained by questionnaire [see Gouveia *et al*. (2017) for a more detailed description of the testing procedures].

### Statistical analyses

For descriptive purposes, we compared sex differences in HRQoL, health-related parameters, and lifestyle variables (using independent samples *t*-tests to compare mean scores and *χ* ^2^ tests to compare sample proportions) and inspected bivariate relations of HRQoL to the health-related parameters and lifestyle variables (BMI, body strength, aerobic endurance, PA, falls, depressive symptoms, and living alone) for the overall sample (using Pearson’s product-moment correlation coefficients, *r*).

Simultaneously taking all these relations into account, we used multiple regression analysis to examine the independent contribution of the correlates of HRQoL. We additionally entered sex as moderator to investigate differences between men and women regarding these relationships. Finally, analyses were repeated separately for the physical and the mental component of HRQoL to investigate whether findings were HRQoL domain specific.

## Results

### Descriptive statistics

#### Means and standard deviations

As apparent in Table 1, men showed higher averages in HRQoL total score and the physical and the mental component of HRQoL, compared with women, as well as lower BMI, higher aerobic endurance, and a lower proportion of individuals with depressive symptoms, falls, and living alone. No sex differences were found for body strength and PA.

#### Bivariate relations

As presented in Table 2, there were significant negative correlations between HRQoL and BMI, depressive symptoms, falls, and living alone. There were significant positive correlations between HRQoL and body strength, aerobic endurance, and PA.

**Table 2:** Pearson correlations between health-related quality of life (HRQoL) and health-related parameters

HRQoL	PC	MC	Total score
BMI (kg/m^2^)	−0.20***	−0.15***	−0.19***
ACT (*n*)	0.36***	0.29***	0.36***
MWT6 (min)	0.51***	0.37***	0.49***
PA total score (3–15)	0.28***	0.20***	0.27***
Depression (0/1)	−0.19***	−0.22***	−0.22***
Fallers (0/1)	−0.24***	−0.19***	−0.24***
Living alone (0/1)	−0.14***	−0.23***	−0.20***

PC=SF-36 physical component; MC=SF-36 mental component; Total score=SF-36 total score; BMI=body mass index; ACT=arm curl test; MWT6=6-min walk test; PA=physical activity; Fallers (0/1), 0=no falls, 1=falls; Depression (0/1), 0=no depressive symptoms, 1=depressive symptoms; Living alone (0/1), 0=living with others, 1=living alone. ***P<0.001.

### Examining sex-specific correlation patterns of HRQoL

Simultaneously considering all relations in the multiple regression model, BMI (*β*=−0.15, *P*=0.001), body strength (*β*=0.21, *P*<0.001), aerobic endurance (*β*=0.29, *P*<0.001), PA (*β*=0.11, *P*=0.007), depressive symptoms (*β*=−0.19, *P*<0.001), falls (*β*=−0.19, *P*<0.001), and living alone (*β*=−0.16, *P*<0.001) were all significantly related to HRQoL total score.

We further investigated whether these relation patterns differed between men and women. Thereby, considering sex as moderator in the multiple regression model, we found a significant interaction of sex with PA on HRQoL total score (*β*=0.08, *P*=0.037). Interestingly, this seemed to account for all previously observed relations of HRQoL to BMI, body strength, aerobic endurance, depressive symptoms, falls, and living alone, also including the initially observed bivariate sex difference in HRQoL, which all did not remain significant (*β*s<0.07, *P*s>0.103). The only significant relation of HRQoL total score remaining was to PA (*β*=0.12, *P*=0.004). We did not find any other significant interactions of sex with the predictors (*β*s<0.06, *P*s>0.138).

To investigate the differential sex patterns in more detail, we inspected the relations of the predictors with HRQoL total score in multiple regressions separately for men and women. Interestingly, PA was related to HRQoL only in men (*β*=0.21, *P*<0.001), but not in women (*β*<0.01, *P*=0.882). Together with the previously reported interaction effect, this suggests that the positive relation of HRQoL total score to PA was significantly higher in men compared with women.

Finally, we further investigated whether these sex-specific relation patterns were universal or whether they differed between the physical and the mental component of HRQoL. Thereby, repeating analyses separately for the two HRQoL components revealed highly similar relation patterns for the physical component of HRQoL, with PA being the only significant correlate of physical HRQoL (*β*=0.12, *P*=0.003) and a significant interaction of sex with PA on physical HRQoL (*β*=0.08, *P*=0.040) as well as PA being related to physical HRQoL only in men (*β*=0.22, *P*<0.001), but not in women (*β*=0.02, *P*=0.717). In contrast, for the mental HRQoL component there was no significant interaction of sex with the predictors (*β*s<0.07, *P*s>0.105).

## Discussion

In this study, higher scores of general HRQoL have been found in men, compared with women. These results are in line with those reported in several samples of older adults (Skar *et al*., 2014; Olmedo-Alguacil *et al*., 2016). Differences between men and women have also been identified in our study regarding both the physical and mental components of HRQoL. This finding is consistent with previous results reported by Orfila *et al*. (2006), in a sample of Spanish older adults assessed by the Nottingham Health Profile.

To explain those sex differences in HRQoL, considering sex as moderator in the multiple regression model, there was a significant interaction of sex with PA on HRQoL total score. The only significant relation of HRQoL remaining was to PA. Additional analyses revealed that PA was only significantly related to HRQoL in men, but not in women. Together with the aforementioned interaction effect, this suggests that, in older adults, PA plays a stronger part in HRQoL in men than in women, and this differential relation may likely explain the higher scores of HRQoL in men.

Assessing HRQoL in a large sample of Spanish older adults, Orfila *et al*. (2006) described the direct effect of sex on HRQoL as very small and non-significant, when controlled for other predictors in a regression model, and argued that, when considering other predictors, including chronic conditions and functional capacity, the effect of sex is indirect or might not even exist. Interestingly, given our observation that considering sex as moderator in the multiple regression model seemed to account for all the initially observed bivariate sex difference in HRQoL suggests that the pathways of indirect effects postulated by Orfila *et al*. (2006) may also concern differential relations of HRQoL to PA (which remained the only significant factor in the regression model).

In regard to the question whether sex-specific relation patterns differed between the physical and the mental component of HRQoL, our findings revealed also for the physical component of HRQoL (as discussed for overall HRQoL before) a significant interaction of sex with PA, and a significant relation between PA and physical HRQoL only in men, while such patterns were not observed for the mental HRQoL component. This finding suggests that sex differences in the relation of PA to HRQoL may only be present for the physical component of HRQoL. This seems reasonable given the particular role of physical and fitness factors for physical HRQoL in older adults (in contrast to mental HRQoL; Gouveia *et al*., 2017).

The main strength of the present study is the representative and large sample of older men and women from the Autonomous Region of Madeira, which allows the generalizability of the findings in this population, therefore supporting the design of regional policies and tailored interventions related to quality of life and PA promotion.

With respect to the latter notions, as a limitation for establishing cause-effect relationships between HRQoL and the identified correlates we acknowledge the cross-sectional design of this population-based study. In addition, PA was assessed by questionnaires. Further research should be developed using objective assessments. Yet, besides the variability of HRQoL predictors accounted for in this study, with higher relevance given to potentially modifiable factors of HRQoL, other variables should be taken into account in future research, in order to better explain HRQoL in older adults, in both men and women.

In conclusion, the present study suggests that interventions to improve HRQoL in older men should include PA promotion. In addition, the present study reinforces the need for further research. A gap in the available knowledge has been identified, particularly regarding the predictors of HRQoL and its differential relations in older men and women. Thus, in addition to a multidimensional approach, sex differences should be accounted for when designing interventions for improving older adults’ HRQoL, particularly when addressing physical HRQoL. Further comprehensive and longitudinal research is needed to enhance knowledge on this topic, as basis for the regional policies and tailored and sex-specific interventions.

## Financial Support

Technical assistance in data collection was supported by the Madeira Regional Government and Regional Secretary of Education and Culture. This study was supported by a doctoral degree grant from the Portuguese national funding agency for science, research and technology (Reference: SFRH/BD/ 29300/2006). A.I. and M.K. acknowledge the support from the Swiss National Center of Competences in Research LIVES – Overcoming vulnerability: life course perspectives, which is financed by the Swiss National Science Foundation (grant number: 51NF40-160590).

## Conflicts of Interest

None.

## Ethical Standards

All procedures were in accordance with the ethical standards of an institutional research committee and with the 1975 Helsinki declaration and its later amendments. Informed consent was obtained from all individual participants included in the study.

## References

[ref1] American College of Sports Medicine (2006) ACSM’s guidelines for exercise testing and prescription, seventh edition Philadelphia, PA: Lippincott Williams & Wilkins.

[ref2] Baecke J, Burema J Frijters J (1982) A short questionnaire for the measurement of habitual physical activity in epidemiological studies. The American Journal of Clinical Nutrition 36, 936–942. 7137077 10.1093/ajcn/36.5.936

[ref3] Choi M, Lee M, Lee MJ Jung D (2017) Physical activity, quality of life and successful ageing among community-dwelling older adults. International Nursing Review 64, 396–404. 28837231 10.1111/inr.12397

[ref4] Dale CE, Bowling A, Adamson J, Kuper H, Amuzu A, Ebrahim S, Casas JP Nüesch E (2013) Predictors of patterns of change in health-related quality of life in older women over 7 years: evidence from a prospective cohort study. Age and Ageing 42, 312–318. 23537589 10.1093/ageing/aft029

[ref5] Dhana K, Berghout MA, Peeters A, Ikram MA, Tiemeier H, Hofman A, Nusselder W, Kavousi M Franco OH (2016) Obesity in older adults and life expectancy with and without cardiovascular disease. International Journal of Obesity 40, 1535–1540. 27163746 10.1038/ijo.2016.94

[ref6] Giuli C, Papa R, Bevilacqua R, Felici E, Gagliardi C, Marcellini F, Boscaro M, De Robertis M, Mocchegiani E, Faloia E Tirabassi G (2014) Correlates of perceived health related quality of life in obese, overweight and normal weight older adults: an observational study. BioMed Central Public Health 15, 14–35. 10.1186/1471-2458-14-35 PMC3898396 24428944

[ref7] Glintborg D, Nielsen TL, Wraae K, Hougaard D, Gudex C, Brixen K Andersen M (2014) The relationship between health-related quality of life, obesity and testosterone levels in older men. Age and Ageing 43, 280–284. 24375324 10.1093/ageing/aft203

[ref8] Gouveia ERQ, Gouveia BR, Ihle A, Kliegel M, Maia JA, Badia SB Freitas DL (2017) Correlates of health-related quality of life in young-old and old–old community-dwelling older adults. *Quality of Life Research* 26, 1561–1569. 10.1007/s11136-017-1502-z. 28110442

[ref9] Haider S, Luger E, Kapan A, Titze S, Lackinge C, Schindle KE Dorner TE (2016) Associations between daily physical activity, handgrip strength, muscle mass, physical performance and quality of life in prefrail and frail community-dwelling older adults. *Quality of Life Research* 25, 3129–3138. 10.1007/s11136-016-1349-8. PMC5102974 27363692

[ref10] Hajek A, Brettschneider C, Lange C, Posselt T, Wiese B, Steinmann S, Weyerer S, Werle J, Pentzek M, Fuchs A, Stein J, Luck T, Bickel H, Mösch E, Wolfsgruber S, Heser K, Maier W, Scherer M, Riedel-Heller SG König HH (2016) Gender differences in the effect of social support on health-related quality of life: results of a population-based prospective cohort study in old age in Germany. Quality of Life Research 25, 1159–1168. 26506992 10.1007/s11136-015-1166-5

[ref11] Hambleton P, Keeling S McKenzie M (2009) The jungle of quality of life: mapping measures and meanings for elders. Australasian Journal on Ageing 28, 3–6. 19243368 10.1111/j.1741-6612.2008.00331.x

[ref12] Hart PD (2016) Sex differences in the physical inactivity and health-related quality of life relationship among rural adults. Health Promotion Perspectives 6, 185–189. 10.15171/hpp.2016.30. 27766235 PMC5071785

[ref13] Hawton A, Green C, Dickens AP, Richards SH, Taylor RS, Edwards R, Greaves CJ Campbell JL (2011) The impact of social isolation on the health status and health-related quality of life of older people. Quality of Life Research 20, 57–67. 20658322 10.1007/s11136-010-9717-2

[ref14] Katz S Calasanti T (2015) Critical perspectives on successful aging: does it “appeal more than it illuminates”? Gerontologist 55, 26–33. 24747713 10.1093/geront/gnu027 PMC4986584

[ref15] Noh J-W, Kim J, Park J, Kim H-J Kwon YD (2015) Gender difference in relationship between health-related quality of life and work status. PLoS ONE 10, e0143579. 26629811 10.1371/journal.pone.0143579 PMC4667923

[ref16] Olivares PR, Gusi N, Prieto J Hernandez-Mocholi MA (2011) Fitness and health-related quality of life dimensions in community-dwelling middle aged and older adults. Health and Quality of Life Outcomes 22, 117. 10.1186/1477-7525-9-117 PMC3286398 22192520

[ref17] Olmedo-Alguacil MM, Ramírez-Rodrigo J, Villaverde-Gutiérrez C, Sánchez-Caravaca MA, Ferrándiz EA Ruiz-Villaverde A (2016) Health-related quality of life, gender, and culture of older people users of health services in the multicultural landscape of the city of Ceuta (Spain): a cross-sectional study. Journal of Transcultural Nursing 27, 603–610. 26220887 10.1177/1043659615597042

[ref18] Olsson SJG, Börjesson M, Ekblom-Bak E, Hemmingsson E, Hellénius M-L Kallings LV (2015) Effects of the Swedish physical activity on prescription model on health-related quality of life in overweight older adults: a randomised controlled trial. BMC Public Health 15, 687. 26193882 10.1186/s12889-015-2036-3 PMC4509721

[ref19] Ono R, Hirata S, Yamada M, Nishiyama T, Kurosaka M Tamura Y (2007) Reliability and validity of the Baecke physical activity questionnaire in adult women with hip disorders. BMC Musculoskeletal Disorders 8, 61. 17610746 10.1186/1471-2474-8-61 PMC1931597

[ref20] Orfila F, Ferrer M, Lamarca R, Tebe C, Domingo-Salvany A Alonso J (2006) Gender differences in health-related quality of life among the elderly: the role of objective functional capacity and chronic conditions. Social Science & Medicine 63, 2367–2380. 16884840 10.1016/j.socscimed.2006.06.017

[ref21] Rejeski WJ Mihalko SL (2001) Physical activity and quality of life in older adults. The Journals of Gerontology. Series A, Biological Sciences and Medical Sciences 56, 23–35. 10.1093/gerona/56.suppl_2.23 11730235

[ref22] Ribeiro JP (2005) O importante é a saúde: estudo de adaptação de uma técnica de técnica de avaliação do estado de saúde SF-36. Lisboa: Merck Sharp & Dolme.

[ref23] Rikli RE Jones CJ (2013) Senior fitness test manual. Development and validation of a functional fitness test for community-residing older adults, second edition Champaign, IL: Human Kinetics.

[ref24] Sivertsen H, Bjørkløf GH, Engedal K, Selbæk G Helvik AS (2015) Depression and quality of life in older persons: a review. Dementia and Geriatric Cognitive Disorders 40, 311–339. 26360014 10.1159/000437299

[ref25] Skar L, Juuso P Sodeberg S (2014) Health-related quality of life and sense of coherence among people with obesity: important factors for health management. *Sage Open Medicine* 2, 1–8. 10.1177/2050312114546923. PMC4607204 26770736

[ref26] Stenhagen M, Ekström H, Nordell E Elmståhl S (2014) Accidental falls, health-related quality of life and life satisfaction: a prospective study of the general elderly population. Archives of Gerontology and Geriatrics 58, 95–100. 23993268 10.1016/j.archger.2013.07.006

[ref27] Takata Y, Ansai T, Soh I, Awano S, Yoshitake Y, Kimura Y, Sonoki K, Kagiyama S, Yoshida A, Nakamichi I, Hamasaki T, Torisu T, Toyoshima K Takehara T (2010) Quality of life and physical fitness in an 85-year-old population. Archives of Gerontology and Geriatrics 50, 272–276. 19419777 10.1016/j.archger.2009.04.005

[ref28] Thiem U, Klaaßen-Mielke R, Trampisch U, Moschny A, Pientka L Hinrichs T (2014) Falls and EQ-5D rated quality of life in community-dwelling seniors with concurrent chronic diseases: a cross-sectional study. Health and Quality of Life Outcomes 12, 2. 24400663 10.1186/1477-7525-12-2 PMC3895701

[ref29] Wanderley FA, Silva G, Marques E, Oliveira J, Mota J Carvalho J (2011) Associations between objectively assessed physical activity levels and fitness and self-reported health-related quality of life in community-dwelling older adults. Quality of Life Research 20, 1371–1378. 21380765 10.1007/s11136-011-9875-x

[ref30] Ware JE Sherbourne CD Jr (1992) The MOS 36-item short-form health survey (SF-36). I. Conceptual framework and item selection. Medical Care 30, 473–483. 1593914

